# Rest for Consumptives

**Published:** 1871-03

**Authors:** 


### "REST FOR CONSUMPTIVES."

One of the most beautiful of the many magnificent charities of liberal New York, the largest-hearted city of the world, is an institution, a year old, called "The House of Rest for Consumptives," where those who have no money can find a comfortable home, if there is any vacancy. To be doomed to certain death, to know that it is an inevitable event, to occur only after weary weeks and months of insupportable wasting cough, and restless nights, and not a pillow for the head, nor a cover for the body, nor a single penny for medicine, or a morsel of food, has scarcely its equal for the terrible; and yet there are loving, thoughtful, and human hearts in New York, who have had a care for such, whose names we are glad to record in the following statement. Reader, if you have a single dollar to spare, send it on right away, and the angel of goodness will make a note of it to be forwarded up to heaven.

The first anniversary of the founding of the House of Rest for Consumptives was held lately, at the House, at Tremont, N. Y. About 150 persons were present, among whom were Bishops Potter and Littlejohn. The annual report was read by the President, Mr. Henry J. Cammann. Through the agency of Miss Bogle, the lady now in charge, the present Board of Trustees was organized in October, a year ago. A building, with about one and a half acres of ground, was obtained at Tremont, and on the 1st of November the House was opened for the admission of patients. The Institution was supported by voluntary contributions from surrounding churches, and many of the articles of furniture were thus received. During the past year, about $10,000 has been expended; 38 patients (21 men, 17 women) have been admitted; 14 have been dismissed, most of them much benefited; 12 have died, and 12 are now in the care of the House.

